# Knowledge, attitudes, and practices of parents toward sexuality education for primary school children in China

**DOI:** 10.3389/fpsyg.2023.1096516

**Published:** 2023-02-01

**Authors:** Wenjing Zhang, Yuzhi Yuan

**Affiliations:** ^1^Beijing Academy of Educational Sciences, Beijing, China; ^2^School of Education, Renmin University of China, Beijing, China

**Keywords:** sexuality education, influencing factors, primary schools, parents, China

## Abstract

This cross-sectional study provides insight into the perceptions of Chinese parents of primary school children with respect to sexuality education. A sample of 19,745 parents was surveyed using an online questionnaire in Beijing, Tianjin, and Hebei, China. SPSS version 23.0 was used for data analysis. In this study, nearly 90% of parents had positive attitudes toward the sexuality education of children in primary schools. However, Chinese parents had limited knowledge of sexuality education. More than 60% of parents were unaware of the sexuality questions that children may encounter at different ages and did not have any accurate information on child sexual abuse prevention education. Although ~ 70% of parents (both fathers and mothers) reported that they had used television and Internet resources to talk with their children about sexuality and 63% reported that they had read books with their children about sexuality, < 30% reported using appropriate terminology with their children on sexuality education and only 40% reported talking to their children about sexuality comfortably. The multivariate linear regression equation showed some factors associated with the practices of parents in sexuality education, including parental knowledge, attitudes, their experience in receiving sex education in childhood, and their educational level. The findings from this study suggest that it is important to develop culturally relevant training programs for parents of primary schools in Chinese society. The implications and limitations of these findings are discussed.

## Introduction

Sexuality education is one of the most important aspects of education for children. The international technical guidance of the United Nations Educational, Scientific, and Cultural Organization (UNESCO) on sexuality education emphasizes the need for comprehensive sexuality education (CSE) programs that aim to equip children and young people with the knowledge, skills, attitudes, and values to make responsible choices about their sexual and social relationships in the world (UNESCO, 2018). CSE programs are scientifically accurate, culturally and age-appropriate, gender-sensitive, and life skills-based. Sexuality education should not be assumed to begin in adolescence. Sexuality begins at birth, and talking about sex and sexuality with children should be a continuous process. Sexuality education if started early in childhood may help children develop a sense of themselves and their bodies while strengthening their self-confidence and helping them take charge of their lives (UNESCO, 2018). Sexuality education is critical in the development of a healthy life; it is both formal (e.g., school-based curricula and health professionals) and informal (e.g., parents /caregivers, Internet, and peers).

### Parents play an important part in the sexuality education of children

Parents play a key role in educating their children about sexuality. They could offer sexuality education sequentially and provide time-sensitive information as they receive questions from their children at home (Krauss and Miller, 2012). CSE programs at school can help children and young people maintain sexuality and relationship health in physical, emotional, spiritual, and social adaptation. The impact of CSE lessons at school depends on the support of parents at home, such as clarifying concepts and helping children apply their new knowledge and skills in daily life. Moreover, one of the most important extended environments affecting the learning activities of their children in sexuality is the teaching of parents at home (Morawska et al., 2015). Children and young people often want to learn about sexuality matters from their parents (Turnbulla et al., 2008). Young children are curious about the issues related to sexuality and ask their parents questions about body differences between boys and girls, where babies come from, how babies are made, and so on (Brilleslijper-Kater and Baartman, 2000; Martin and Torres, 2014). When parents talk about sexuality issues with their children at an early age, they have the opportunity to foster safe and healthy attitudes toward sexuality (Morawska et al., 2015). Therefore, the knowledge, attitudes, and practices of parents would have very strong influences on the formation of sound sex-related values in their children.

### Parental knowledge, attitudes, and practices in the sexuality education of children

Previous studies showed that a majority of parents have positive attitudes toward sexuality education at school and have general sexuality knowledge (Mckay et al., 2014; Morawska et al., 2015; Shin et al., 2019). For example, one study in Australia, with a sample of 557 parents of children between the ages of 3 and 10 years, demonstrated that parents felt knowledgeable about sexuality education (Morawska et al., 2015). Another survey of 337 primary school parents conducted in Korea revealed that more than 70% of respondents have the knowledge of the structure and function of sexual organs, pregnancy, and childbirth (Shin et al., 2019).

Research has also shown that few parents discuss and communicate about sexuality with their children. Some studies indicated that few parents could provide adequate and good-quality sexuality education for their children (Jerman and Constantine, 2010; Morawska et al., 2015; Shin et al., 2019). Morawska et al. (2015) found that more than half of the Australian parents did not start up a conversation about sexuality with their children (aged 3–10 years). A recent study in South Korea also showed that two-thirds of parents had never provided sexuality education to their elementary school children (Shin et al., 2019). Scholars and researchers have proposed several reasons for the reluctance of parents to talk about sexuality with their children. These include parents having an inability or unwillingness to discuss topics of a sensitive nature, especially on sexuality (Morawska et al., 2015; Alldred et al., 2016); parents lacking sexuality knowledge and teaching skills to provide education (Morawska et al., 2015; Shin et al., 2019); parents with low self-efficacy or feeling embarrassment (Turnbulla et al., 2008; Morawska et al., 2015); and parents with the fear of destroying the “innocence” of their children (Alldred et al., 2016; Robinson and Davies, 2017; Rudolph and Zimmer-Gembeck, 2018). Sexuality education is effective when parents and their children discuss sex-related issues together. Furthermore, parent-child communication, parental monitoring, and involvement are all beneficial to protecting children from sexual abuse (Wurtele and Kenny, 2010; Rudolph and Zimmer-Gembeck, 2018). Parent-child communication about sexuality in the family has been linked to reductions in risky sexual behaviors in adolescence (Huebner and Howell, 2003; Zhao et al., 2022).

### Sexuality education of children in China

China has long recognized the significance of sexuality education for children. In March 1963, Premier Zhou Enlai stressed the need to promote sexuality education for adolescents as an important element for the healthy growth of the Chinese population on the occasion of the national conference on the hygiene science and technology program (Liu, 1994). However, for a long time, there are neither national guidelines nor a national curriculum for sexuality education in China. In 2008, the Ministry of Education issued a set of health education guidelines for primary and middle schools that contained some reference to sexuality education (Ministry of Education of People's Republic of China, 2008). Recently, child sexual abuse has become a major concern in Chinese society. A meta-analysis found that child sexual abuse prevalence rates are 8.9% for women and 9.1% for men in China (Ma, 2018). Therefore, in 2013 and 2018, the Ministry of Education issued opinions on sexual abuse education prevention for children and adolescents (Ministry of Education of People's Republic of China, 2013, 2018). In the present time, part from sexual abuse, children in China are frequently exposed to sexual messages through films, television, the media, and the Internet. Thus, the importance of quality sexuality education at an early age has been increasingly emphasized in modern Chinese society (Zhang et al., 2013; Liu and Su, 2014).

Although the education policy of China supports primary and middle schools to carry out sexuality education, very few schools officially teach sexuality education courses for children (Liu and Su, 2014). Chinese children, especially primary school students, have limited sexuality knowledge and skills. A survey with 773 primary students (aged 6–14 years) conducted in 12 schools in Zhejiang, Hunan, Shanxi, Sichuan, Ningxia, and Liaoning provinces in China found that primary school students lack sexuality knowledge (Hu et al., 2015). Some studies in China on child sexual abuse prevention education revealed that primary school children lack the knowledge of sexual abuse prevention and self-protection skills (Chen, 2012; Jin et al., 2016).

Although more than 70% of primary school students identify their parents as the main source of sexuality education in China (Hu et al., 2015), there is less research on the sexuality education of the children of primary school parents. In Fuzhou, a city in the southeast of China, one study investigated 209 parents in four primary schools. It was found that < 40% of parents reported their children needed to receive sexuality education at school and that nearly one-half of parents were reluctant to answer questions about the sexuality development of their children (Hu, 2017). Diao surveyed a sample of 179 primary school parents in Chengdu, a city in southwest China. It was reported that more than 90% of parents had positive attitudes toward school-based sexuality education, but only one-third of parents could answer questions about the sexuality development of their children (Diao, 2019).

In the last three decades, it has been found that a series of qualitative or review studies involves the concept of sexuality education, teaching modes, education lessons, resources, and practices in China (Liu and Yuan, 2017; Wu and Zeng, 2020; Zhao et al., 2020). However, there is less empirical and quantitative research on sexuality education. Previous quantitative studies in China that included primary school parents only had small sample sizes, mainly in the south of China. In addition, previous studies showed that there are some impact factors related to parental communication about sexuality with their children. The main related factors include parental characteristics, such as parental gender, educational level, experience in receiving sexuality education in childhood, sexuality knowledge, and attitudes (Mckay et al., 2014; Flores and Barroso, 2017; Robinson and Davies, 2017; Shin et al., 2019), and child characteristics, such as child gender, age, and grade (Flores and Barroso, 2017; Shin et al., 2019).

### The current study

Therefore, the present study had two aims. First, this study aimed to examine knowledge, attitudes, and educative practices toward sexuality education among parents of primary school students in Beijing, Tianjin, and Hebei in China. Second, the study sought to explore whether sexuality communication of Chinese parents with their children is associated with certain “facilitators” such as the parental experience of receiving sexuality education in childhood, parental sexuality knowledge, parental attitudes toward sexuality education, parental educational level, child gender, child grade, and other demographic factors. The findings of this study are expected to be used as baseline data to develop an education program to assist parents in discussing sexuality with their children in China.

## Materials and methods

### Procedures and participants

A cross-sectional survey was conducted among a convenience sample of primary school parents in Beijing, Tianjin, and Hebei. Participating parents were from six primary schools located in Beijing, four schools in Tianjin, and five schools in Hebei. An online questionnaire was used to collect data from participating parents. First, a link to the questionnaire was sent to the heads of participating schools. Second, school head teachers were asked to forward the link to parents to promote participation. Third, parents accessed the survey online and were presented with an information page about the study as well as a consent page, such as the voluntary nature of their participation, the anonymity and privacy of their responses, and so on. Participants could exit the online survey at any time. The questionnaire was available online for 4 months, from September to December 2018.

Of the 28,155 enrolled primary students, 21,679 adults responded (77.0%), with only one adult permitted to respond per family. Among the 21,679 online questionnaires returned, 277 questionnaires were rejected due to the fact that the respondents missed over one-third of the questions (199) or gave fictitious or inconsistent (e.g., The answer for more than two-thirds of the items in the questionnaire was the same option, such as option A, option B, and so on; and more than one-third of the items had been given two answers, but two answers were inconsistent) answers (78). The data analyses in the current study were restricted to respondents of mothers and fathers only. The final participants were 19,745 (92.3%) parents, including 15,217 mothers (77.1%, *M* age = 35.96, *SD* = 4.42) and 4,528 fathers (22.9%, *M* age = 37.37, *SD* = 4.87). Their children were aged 6–13 years (*M* age = 8.77, *SD* = 1.78).

### Measures

#### Parent questionnaire

The parental questionnaire was developed based on a series of published studies (Chen and Chen, 2005; Dake et al., 2014; Morawska et al., 2015; Robinson et al., 2017; Depauli and Plaute, 2018; Rudolph and Zimmer-Gembeck, 2018; UNESCO, 2018; Shin et al., 2019) and included 15 items on knowledge, attitudes, and practices regarding sexuality education of children.

The knowledge subscale included five items, including knowledge of correct terminology for genitalia, daily sexual healthcare, child sexual development, and sexuality questions, that may be encountered at different ages of development of children, and accurate information on child sexual abuse prevention education, “*e.g., that do you know the correct terminology for genitalia”*. Response options were “*yes,” “no,”* or “*unsure”* for each item. “Yes” responses scored 1, while “no” and unsure responses scored 0. The scores for each item were summed for a total knowledge score (*range* = *0–5*). A brief attitudes subscale consists of three items that ask whether parents agree or disagree with the aspects of sexuality education for children in primary school (0–3). The practices subscale included seven items. These seven questions were asked about parental communication with their child about sexuality. Examples of questions in this section included the following: “Did you use appropriate terminology with your child in the process of sexuality education?” “Did you encourage your child to share their thoughts and feelings about sexuality?” and “Did you give brochures or other materials to your child to help them learn about sexuality?” Response options were “*yes,” “no,”* or “*unsure”* for each item. “Yes” responses scored 1, while “no” and unsure responses scored 0. The scores for each item were summed for a total practice score (*range* = *0–7*). Internal consistency analyses of subscales of knowledge, attitudes, and practices produced alpha levels of 0.83, 0.90, and 0.80, respectively.

#### Demographic form

A demographic form was designed to gather background information about parents. Four items gathered demographic information about themselves, including gender (fathers/mothers), age, education qualifications (junior high school or less/senior high school/college degree/bachelor degree or high), and their childhood sexuality education history. Parents were asked about their own experience of receiving sexuality education in childhood: “In your childhood, did you ever get the information about sexuality education from your parents or other family members?” and “In your childhood, did you ever get the information about sexuality education at school?” (response options: yes, no, or unsure). Other four items were used to gather the characteristics of their children, including child gender (boys/girls), child grade, child age, and only one child or not (yes/no).

The whole questionnaire was reviewed by two psychologists, two sexuality education professionals, and two educational researchers. These interviews and their evaluation were discussed. Then, their suggestions and proposals for improvement were taken into account in the design of the final questionnaire. The final questionnaire was piloted with ten parents of primary students, including four fathers and six mothers, to determine whether they were able to comprehend the instructions and items of the questionnaire. No changes were made to the final questionnaire following the current study.

### Data analyses

All data were analyzed using the SPSS 23.0 software. First, descriptive statistics were used to report the demographic characteristics of parents and their knowledge, attitudes, and practices toward the sexuality education of children in primary schools. Second, a series of variance analyses were conducted to investigate the associations between scores of parents on sexuality knowledge, attitudes, and educative practices and their sociodemographic characteristics variables. Finally, a stepwise multivariate linear regression was conducted to find the correlated factors of parental practices toward the sexuality education of children.

## Results

### Demographic characteristics of parents

The present sample included 19,745 parents. Table 1 shows the frequency distribution of parental demographic variables.

**Table 1:** Characteristics of the parents involved in the study (*N* = 19,745).

**Characteristics**		***n* (%)**
Living region	Beijing	5,639 (28.6%)
	Tianjin	2,681 (13.6%)
	Hebei	11,425 (57.9%)
Gender	Mothers	15,217 (77.1%)
	Fathers	4,528 (22.9%)
Mothers' educational level	Junior high school or less	4,480 (22.7%)
	Senior high school	5,526 (28.0%)
	College degree	4,710 (28.6%)
	Bachelor degree or above	5,029 (25.5%)
Fathers' educational level	Junior high school or less	4,338 (22.0%)
	Senior high school	6,041 (30.6%)
	College degree	4,609 (23.3%)
	Bachelor degree or above	4,757 (24.1%)
Only-one child	Yes	9,218 (46.7%)
	No	10,527 (53.3%)
Child gender	Girls	9,408 (47.6%)
	Boys	10,337 (52.4%)
Child grade	Grade 1	3,763 (19.1%)
	Grade 2	3,005 (15.2%)
	Grade 3	3,562 (18.0%)
	Grade 4	3,407 (17.3%)
	Grade 5	2,896 (14.7%)
	Grade 6	3,112 (15.8%)

### History of sexuality education for parents

Parents were asked about their own experiences of receiving sexuality education in childhood. Less than 7% (6.6%; fathers: 5.7% and mothers: 6.8%) of respondents reported that they did receive sexuality education from their family in childhood, and ~10% (10.3%; fathers: 10.8% and mothers: 10.1%) of respondents reported that they received sexuality education from the school in childhood. The proportion of parents who received sexuality education in childhood at school was significantly higher than that at home (χ^2^ = 3,524.45, *p* < 0.001). More mothers than fathers received family sexuality education in childhood (χ^2^ = 7.13, *p* < 0.01). There was no gender difference in the parental history of receiving sexuality education at school (*p* > 0.05).

### Knowledge, attitudes, and practices of parents toward sexuality education of children

Correct responses of parents to sexuality knowledge questions of children are summarized in Table 2. More than 80% of the parents responded that they have the knowledge of the correct terminology for genitalia and the knowledge about daily sexual healthcare. In other respects, the sexuality knowledge of parents is lacking. Nearly half of the participating parents did not know about sexual development and behavior across childhood, while almost three-fifths did not know about sexuality questions that children may encounter at different ages and did not have any accurate information on child sexual abuse prevention education. On average, parents of primary school students had 60% of sexuality knowledge (*M* = 3.02, *SD* = 1.54).

**Table 2:** Parental knowledge of children sexuality education (*N* = 19,745).

**Items**	**Answered correctly (%)**		

	**Mothers**	**Fathers**	**Total**
1. Correct terminology for genitalia	84.9	90.0	86.1
2. Daily sexual health care	86.7	82.2	85.7
3. Sexual development and behavior across childhood	52.3	49.6	51.7
4. Questions about sexuality that may be encountered at different ages of children development	39.1	38.6	39
5. Accurate information on child sexual abuse prevention education	40.5	37.1	39.7

As shown in Table 3, ~ 90% of parents agreed that sexuality education should be offered to primary school students, and primary school teachers should provide sexuality education to their students. Moreover, the great majority of parents were willing to join school sexuality education programs. In short, parents had positive attitudes toward sexuality education in primary schools (*M* = 2.61, *SD* = 0.87).

**Table 3:** Parental attitudes toward sexuality education of children (*N* = 19,745).

**Items**	**Answered “agree” (%)**		

	**Mothers**	**Fathers**	**Total**
1. Primary school students should receive sexuality education?	88	84	87.1
2. Primary school teachers should provide their students with sexuality education	87.5	85.4	87.0
3. Parents should join in school sexuality education programs?	87.9	84.6	87.1

As a whole, 87.1% of parents had provided at least one form of sexuality education at home. However, only 13.6% of the parents answered “yes” to all seven items of sexuality education practices. Approximately 70% of parents reported that they had used television and Internet resources to talk with their children about sexuality appropriately, and nearly two-thirds of parents reported that they had read books with their children about sexuality. More than half of the parents had encouraged their children to share thoughts and feelings about sexuality and had given brochures or other materials to their children to help them learn about sexuality. Using appropriate terminology with their children in the process of sexuality education and talking to their children about sexuality comfortably were noticeably less common. Table 4 shows that the great majority of parents did not respond confidently to questions from their children about sexuality topics. On average, parents of primary school students had limited practices of sexuality education of children at home (*M* = 3.39, *SD* = 2.27).

**Table 4:** Parental practices on sexuality education of children (*N* = 19,745).

**Items**	**Answered “yes” (%)**		

	**Mothers**	**Fathers**	**Total**
1. Responded confidently to a question about a sexuality topic	38.1	46.1	40
2. Used appropriate terminology with my child in the process of sexuality education	27.7	37.2	29.8
3. Talked to my child about sexuality comfortably	30.2	34.6	31.2
4. Encouraged my child to share their thoughts and feelings about sexuality	54.9	53.0	54.5
5. Read books with my child about sexuality	64.0	60.8	63.3
6. Made use of the television and the internet resources to my child about sexuality appropriately	70.3	66.9	69.5
7. Gave brochures or other materials to my child to help them learn about sexuality	50.5	53.4	51.1

### Associations between demographic factors and knowledge, attitudes, and educative practices toward sexuality education of children

Associations between demographic factors of parents and their knowledge, attitudes, and practices toward sexuality education of children are summarized in Table 5. The results showed that the gender of parents was associated with differences in the scores of sexuality knowledge, attitudes, and educative practices. The total score of knowledge of the mother was significantly higher than that of the father (mothers: 3.04 ± 1.54 vs. fathers: 2.98 ± 1.55; *F* = 5.21, *p* < 0.05). Similarly, the score of attitudes of the mother toward sexuality education was significantly higher than that of the father (mothers: 2.63 ± 0.84 vs. fathers: 2.54 ± 0.65; *F* = 40.64, *p* < 0.01). However, the score of practices of the father in the sexuality education of children was significantly higher than that of the mother (fathers: 3.52 ± 2.38 vs. mothers: 3.63 ± 2.23; *F* = 17.98, *p* < 0.01). Further analysis showed that mothers had a higher percentage than fathers in two items “read books with my child about sexuality” (mothers: 64% vs. fathers: 60.8%, χ^2^ = 15.03, *p* < 0.001) and “made use of the television and the Internet resources to my child about sexuality appropriately” (mothers: 70.3% vs. fathers: 66.9%, χ^2^ = 19.22, *p* < 0.001).

**Table 5:** Associations between demographic factors and parental sexuality knowledge, attitudes, and educative practices (*N* = 19,745).

**Variables**	**Categories**	**Sexuality knowledge (0–5)**		**Sexuality attitudes (0–3)**		**Educative practices (0–7)**	

		***M*** **(SD)**	***F***	***M*** **(SD)**	***F***	***M*** **(SD)**	***F***
Living region	Beijing	3.05 (1.54)	7.35^***^	2.67 (0.80)	16.46^**^	3.49 (2.30)	10.43^***^
	Tianjin	3.10 (1.57)		2.62 (0.87)		3.47 (2.32)	
	Hebei	2.99 (1.54)		2.58 (0.90)		3.33 (2.23)	
Gender	Mothers	3.04 (1.54)	5.20^*^	2.63 (0.84)	40.64^***^	3.36 (2.23)	17.98^***^
	Fathers	2.98 (1.54)		2.54 (0.95)		3.52 (2.38)	
Mothers' educational level	Junior high school or less	2.64 (1.63)	171.10^***^	2.46 (1.02)	96.26^***^	2.91 (2.20)	179.78^***^
	Senior high school	2.98 (1.54)		2.57 (0.91)		3.22 (2.21)	
	College degree	3.09 (1.48)		2.67 (0.80)		3.49 (2.25)	
	Bachelor degree or above	3.34 (1.44)		2.74 (0.69)		3.93 (2.28)	
Fathers' educational level	Junior high school or less	2.63 (1.63)	162.13^***^	2.50 (0.99)	64.85^***^	2.93 (2.19)	169.09^***^
	Senior high school	3.00 (1.54)		2.56 (0.93)		3.22 (2.24)	
	College degree	3.11 (1.48)		2.67 (0.79)		3.52 (2.25)	
	Bachelor degree or above	3.33 (1.54)		2.72 (0.72)		3.92 (2.27)	
Only-one child	Yes	3.11 (1.51)	53.82^***^	2.66 (0.81)	52.43^***^	3.56 (2.27)	94.25^***^
	No	2.95 (1.56)		2.57 (0.92)		3.25 (2.25)	
Child gender	Girls	3.11 (1.53)	64.81^***^	2.65 (0.82)	34.35^***^	3.42 (2.24)	3.03
	Boys	2.94 (1.55)		2.58 (0.91)		3.37 (2.29)	
Child grade	Grade 1	2.86 (1.48)	19.08^***^	2.58 (0.92)	1.32	3.32 (2.26)	3.29^**^
	Grade 2	3.01 (1.54)		2.62 (0.88)		3.45 (2.25)	
	Grade 3	2.95 (1.55)		2.61 (0.87)		3.32 (2.24)	
	Grade 4	3.05 (1.56)		2.62 (0.85)		3.37 (2.27)	
	Grade 5	3.15 (1.54)		2.62 (0.84)		3.46 (2.28)	
	Grade 6	3.16 (1.56)		2.61 (0.87)		3.48 (2.30)	
Received sexuality education	Yes	4.21 (1.27)	859.80^***^	2.84 (0.55)	100.02^***^	5.11 (2.05)	833.45^***^
as a child at home	No	2.94 (1.52)		2.60 (0.88)		3.27 (2.23)	
Received sexuality education	Yes	3.87 (1.39)	711.36^***^	2.79 (0.63)	100.40^***^	4.59 (2.20)	656.54^***^
as a child at school	No	2.92 (1.53)		2.59 (0.89)		3.26 (2.23)	

^*^p < 0.05; ^**^p < 0.01; ^***^p < 0.001.

The living region of the parents was associated with knowledge, attitudes, and practices toward the sexuality education of children. Further analysis demonstrated that parents in Beijing and Tianjin had higher scores of sexuality knowledge and practices than that in Hebei (*p* < 0.05), while parents in Beijing and Tianjin had similar levels of knowledge and practices. The results also found that parents in Beijing had more positive attitudes toward the sexuality education of children than that in Tianjin and Hebei (*p* < 0.05), while parents in Tianjin and Hebei had no living region differences in scores of attitudes.

The level of education of parents was positively associated with their sexuality knowledge, attitudes, and educative practices. As shown in Table 5, with the increase in the level of education of parents, their scores on sexuality education knowledge, attitudes, and practices of children were also increasing. Parents who had a bachelor's degree or above scored highest on the knowledge, attitudes, and practice toward sexuality education of children. The experiences of parents receiving sexuality education in childhood were also analyzed. Parents who had experiences receiving sexuality education in childhood at home or school showed significantly higher knowledge scores, more positive attitudes, and more active education practices with their children on sexuality than those who had not (see Table 5).

It is also noteworthy that child gender was associated with parental knowledge and attitudes toward sexuality education. Compared to parents of boys, parents of girls scored higher on sexuality knowledge and attitudes. Regarding the educative practices of parents, there was no significant difference between boys and girls. Moreover, the findings showed that parents who had only one child scored significantly higher scores on knowledge, attitudes, and practices toward sexuality education than those who had not. Child grade was related to the scores of parental sexuality knowledge and practices. Further analysis showed that parents whose children were in grades 5 and 6 had significantly higher sexuality knowledge scores and had more active practices than those whose children were in other grades (see Table 5). Regarding the attitudes of parents toward sexuality education, there was no significant difference by child grade.

### Regression analyses

The stepwise multivariate linear regression was used to explore the associations between the scores of the father (or mother) on sexuality education practices and nine independent variables, including parental knowledge, parental attitudes, parental history of receiving sexuality education at home in childhood, parental history of receiving sexuality education at school in childhood, parental educational level, parental living region, child grade, and only one child.

The multivariate linear regression showed that, among all these variables, the knowledge of the father (*B* = 0.50, *SE B* = 0.02, *p* < 0.00), the attitude of the father (*B* = 0.63, *SE B* = 0.03, *p* < 0.001), history of the father receiving sexuality education at home in childhood (*B* = 0.96, *SE B* = 0.15, *p* < 0.001), history of the father receiving sexuality education at school in childhood (*B* = 0.43, *SE B* = 0.11, *p* < 0.001), and educational level of the father (*B* = 0.16, *SE B* = 0.03, *p* < 0.01) were the significant factors for education practices of the father. The five variables together accounted for 24.4% of the variance in scores on practices of the father toward sexuality education of children (*F* = 183.48, *p* < 0.001, *R*^2^ = 0.24).

There are also five significant factors for the educative practices of the mother. The multivariate linear regression showed that these significant factors included knowledge of the mother (*B* = 0.46, *SE B* = 0.01, *p* < 0.001), the attitude of the mother (*B* = 0.63, *SE B* = 0.02, *p* < 0.001), history of the mother receiving sexuality education at home in childhood (*B* = 0.89, *SE B* = 0.07, *p* < 0.001), history of the mother receiving sexuality education at school in childhood (*B* = 0.50, *SE B* = 0.06, *p* < 0.001), and educational level of the mother (*B* = 0.20, *SE B* = 0.02, *p* < 0.001). The five variables together accounted for 24.9% of the variance in scores on practices of the mother toward sexuality education of children (*F* = 630.38, *p* < 0.001, *R*^2^ = 0.25).

## Discussion

### Knowledge and attitudes of parents toward sexuality education of children

In this study, parents of primary students had gaps in their sexuality knowledge. Although the average sexuality knowledge score was 3.02, the correct rate for some items was very low. Only 39% of parents reported that they had knowledge of the sexuality questions that children may encounter at different ages. Although children commonly ask their parents some sexuality questions (Krauss and Miller, 2012; Martin and Torres, 2014; Hu et al., 2015), parents who lack sexuality knowledge feel embarrassed (Xie et al., 2015; Shin et al., 2019) and may be unable to answer questions from their children in time. Similar to the previous studies done in China (Chen and Chen, 2005; Chen et al., 2007; Xie et al., 2015; Jin et al., 2019), more than 60% of the parents in this study reported that they lacked knowledge about child sexual abuse prevention education. Thus, children may be unable to be protected well and taught accurate information about sexual abuse by their parents (Wurtele and Kenny, 2010).

Most parents (87%) agreed about the importance of sexuality education lessons in primary schools. This finding is consistent with observations in previous studies conducted on US parents of primary school children (~90% in Fisher et al., 2015; 80% in Dake et al., 2014), on Canadian parents (87% in Mckay et al., 2014), and on Australian parents (71% in Robinson et al., 2017) but much higher than the study in Shenzhen province in China 15 years ago (~60% in Wu, 2005). It is suggested that Chinese parents have more positive attitudes toward sexuality education than before. Scholars and researchers advocate that better communication between parents and school teachers could enhance the effect of sexuality education (Robinson et al., 2017). Approximately 90% of parents of primary school students expressed their willingness to join in school-based sexuality education in the current study. This finding suggests that there is substantial support from the majority of parents for implementing sexuality education in primary schools. In future, it is supposed to be a collaborative approach between families and schools on sexuality education in China.

### Practices of parents on sexuality education of children at home

With the help of media (e.g., television and the Internet), parents actively carry out sexuality education at home in China. Almost 70% of parents reported using television and Internet resources to provide sexuality education to their children. The percentage is higher than the previous studies in Australia (Morawska et al., 2015), the United States (Dake et al., 2014), and Korea (Shin et al., 2019). In addition, more than 60% of the parents reported reading books with their children about sexuality, which is a substantial rise from the Australian study which found that < 50% of parents had read books with their children about sexuality (Morawska et al., 2015). At present, the media has become one of the most important sources of sexuality health information in China. Access to media resources could increase motivation and enhance the quality of sexuality education (Lou et al., 2006). Therefore, it is helpful and convenient for parents to discuss sexuality with their children by using the Internet and television.

Chinese parents rarely used correct terminology for genitalia (30%), which is lower than the previous studies with parents of primary school students in Australia (~80% in Morawska et al., 2015) and the United States (~40% in Dake et al., 2014) but much higher than the recent Muslim research in Pakistan (~6% in Nadeem et al., 2021) and the Korean research (~6% in Shin et al., 2019). Parents commonly feel embarrassed or uncomfortable talking to their children about sexuality (Turnbulla et al., 2008). Our study found that less than one-third of the parents had talked to their children comfortably about sexuality, and only two-fifths responded confidently to a question about a sexuality topic. It is much lower than the previous studies in Australia (~75% in Morawska et al., 2015) and Korea (~70% in Shin et al., 2019). The Chinese tradition of sexual conservatism may make it difficult for parents to discuss sexuality topics with their children openly and comfortably (Xie et al., 2015).

### Influencing factors for practices of parents on sexuality education of children

Our study provided a factual basis that parental knowledge and attitudes are influencing factors for their practices on the sexuality education of children. With the increase in parental scores on knowledge and attitudes, their scores on educative practices about sexuality were also increasing. Hence, sexuality education needs to target not just children but also their parents (Robinson et al., 2017). In view of this, scholars and researchers should develop parent-focused sexuality education programs that include more accurate and specific sexuality information to help Chinese parents improve their family communication about sexuality.

Consistent with previous studies (Lee and Kweon, 2013; Flores and Barroso, 2017), the results of our study showed that mother respondents had more knowledge and positive attitudes toward sexuality education of children. However, unlike the previous studies in the United States (Flores and Barroso, 2017) and Korea (Lee and Kweon, 2013), which found that mothers were predominant providers of sexuality education at home, this study showed that fathers were more likely to provide educative practices to their children in China. Fathers also play an important role in sexuality education at home, like mothers. Compared to mothers, fathers may discuss male issues more effectively with their boys (Shin et al., 2019; Zhang et al., 2019). The results of this study suggest that both mothers and fathers of young children should be the target population for sexuality education training programs in China in future.

Despite the vast majority of parents (90%) in the sample having not had received any experience of sexuality education either from their own families or in their school settings, this study still demonstrated that the experience of parents of sexuality education in childhood was associated with better communication about sex-related issues with their child. Consistent with the previous studies (Wight et al., 2006; Jerman and Constantine, 2010; Zhang et al., 2013; Dake et al., 2014), this study also found that parents with higher educational levels were more likely to talk with their children about sexuality. Therefore, attention should be paid to parents with low academic qualifications in future training programs.

### Study limitations

This study had several limitations. The first limitation relates to the purpose-built questionnaire. The scale of knowledge, attitudes, and practices in sexuality education for children was designed to be simple and relatively superficial. While the scales appeared to demonstrate good internal consistency, the results need to be interpreted with caution. To obtain a deeper understanding of the awareness of parents about sexuality education, a more detailed assessment, focus groups, and qualitative interviews need to be conducted in future research. Second, this study used an Internet-based survey that relied on self-reporting. Therefore, the reliability of the data may depend on the sincerity of the answers of respondents. Third, although this study had a sufficient sample size, it was limited to self-reports from a sample of parents only in Beijing, Tianjin, and Hebei in China. Thus, the findings cannot be generalized to the whole of China. Finally, the information collected was cross-sectional; thus, causation between associated factors and parental educative practices in sexuality education cannot be inferred from this study.

## Conclusion

This study concluded that the majority of parents of primary school students had positive attitudes toward sexuality education in China. However, they had limited sexuality knowledge. Less than 40% of the parents knew that there were sexuality questions that children may encounter at different ages and had accurate information on child sexual abuse prevention education. One major benefit of this study lies in the novel investigation that most fathers and mothers had communication specifically with their children about sexuality, with the help of the Internet, television, and books. Moreover, this study documented that parental practices are positively associated with their knowledge and attitudes toward sexuality education of children. Based on these findings, it is important to develop culturally relevant training programs for parents in Chinese society in future. Sexuality education training programs are supposed to help parents learn more accurate knowledge and improve their communication with their young children at home.

## Data availability statement

The datasets presented in this article are not readily available because the data have not been made available on a permanent third-party archive because Beijing academy of educational sciences ruled that we could not post the data. Requests to access the datasets should be directed to the corresponding author (yuanbnu@163.com).

## Ethics statement

The study involving human participants was reviewed and approved by Beijing Academy of Educational Sciences. The participants provided their written informed consent to participate in this study.

## Author contributions

WZ: acquisition of data, conception and design of study, conducting a research and investigation process, and drafting the manuscript. YY: data curation, revising the manuscript critically for important intellectual content, and development or design of methodology. All authors contributed to the article and approved the submitted version.
